# Evaluation of normal swallowing functions by using dynamic high-density surface electromyography maps

**DOI:** 10.1186/s12938-017-0424-x

**Published:** 2017-11-21

**Authors:** Mingxing Zhu, Bin Yu, Wanzhang Yang, Yanbing Jiang, Lin Lu, Zhen Huang, Shixiong Chen, Guanglin Li

**Affiliations:** 1 Chinese Academy of Science (CAS) Key Laboratory of Human-Machine Intelligence-Synergy Systems, Shenzhen Institutes of Advanced Technology, Shenzhen, 518055 China; 2 Designed Intelligence Group, Industrial Design Department, Eindhoven University of Technology, 5612 AZ Eindhoven, The Netherlands; 3 The Rehabilitation Department, Shenzhen Hospital of Southern Medical University, Shenzhen, 518052 China; 4 The Rehabilitation Department, Shenzhen Sixth People’s Hospital, Shenzhen, 518052 China; 5 The Department of Rehabilitation Medicine, Guangzhou Panyu Central Hospital, Guangzhou, 511400 China

**Keywords:** High-density surface electromyography, Energy maps, Muscle contractions, Swallowing effort, Normal swallowing

## Abstract

**Background:**

Swallowing is a continuous process with substantive interdependencies among different muscles, and it plays a significant role in our daily life. The aim of this study was to propose a novel technique based on high-density surface electromyography (HD sEMG) for the evaluation of normal swallowing functions.

**Methods:**

A total of 96 electrodes were placed on the front neck to acquire myoelectric signals from 12 healthy subjects while they were performing different swallowing tasks. HD sEMG energy maps were constructed based on the root mean square values to visualize muscular activities during swallowing. The effects of different volumes, viscosities, and head postures on the normal swallowing process were systemically investigated by using the energy maps.

**Results:**

The results showed that the HD sEMG energy maps could provide detailed spatial and temporal properties of the muscle electrical activity, and visualize the muscle contractions that closely related to the swallowing function. The energy maps also showed that the swallowing time and effort was also explicitly affected by the volume and viscosity of the bolus. The concentration of the muscular activities shifted to the opposite side when the subjects turned their head to either side.

**Conclusions:**

The proposed method could provide an alternative method to physiologically evaluate the dynamic characteristics of normal swallowing and had the advantage of providing a full picture of how different muscle activities cooperate in time and location. The findings from this study suggested that the HD sEMG technique might be a useful tool for fast screening and objective assessment of swallowing disorders or dysphagia.

## Background

Swallowing, a sensorimotor behavior, helps propelling saliva or ingesting bolus from the mouth through the pharynx into the esophagus. It is a complex process comprised by a sequence of highly organized activities involving the brainstem, neural network, and innervations of muscles associated with the oral cavity, pharynx, and esophagus [1, 2]. During swallowing, a high level neuromuscular synergy driven by the skeletal (tongue) muscles and smooth muscles of the pharynx and esophagus are required in order to successfully execute the swallowing sequences [3, 4]. One of the most important physiological events that occur during the pharyngeal stage of swallowing is hyolaryngeal elevation. During the hyolaryngeal elevation, the hyoid and larynx elevate by the contraction of at least three submental muscles (mylohyoid, geniohyoid, and anterior digastric) to protect the airway and to help open the upper esophageal sphincter. Patients with disorders in submental muscles might be exposed to the risk of penetration and or aspiration of bolus materials [5]. The main function of infrahyoid muscles is to depress the hyoid bone and larynx during swallowing, so that the food bolus could be transmitted to the stomach though the esophagus [6], any impairment of these muscles may easily result in disability of food passing. Additionally, from the physiological point of view, EMG activity in the submental muscles most often initiated the swallow whereas the infrahyoid muscle activity most frequently terminated the swallow [7]. Muscle contraction time, muscle strength and muscle synergy all play significant roles in the swallowing process. As a consequence, any problem with these muscles may result in swallowing difficulty known as dysphagia. Dysphagia could lead to different clinical risks such as choking, significant weight loss, and aspiration pneumonia [8, 9], and has been reported to be a common problem of all age groups especially the elderly. Studies showed that approximately 15% of individuals of age 60 or above suffered from dysphagia [10]. Moreover, dysphagia could deteriorate the nutritional status of the patients and thus slow down the recovery rate from particular diseases. Therefore, early diagnoses of dysphagia could be of great importance for their daily life.

The evaluation of the normality of swallowing function plays an important role for the diagnoses of dysphagia. Currently, popular clinical methods for evaluating the swallowing functions in humans include videofluoroscopy [11], endoscopy esophageal manometry [12], ultrasonography [13], acoustic analysis [14, 15], magnetic resonance imaging (MRI) [16, 17], and so on. While these conventional approaches can examine swallowing functions mechanically and/or biomechanically, they would hardly provide electrophysiological information on muscular activities during swallowing. The electrophysiological information of swallowing muscles would be important for exploring the functions of deglutition and for evaluating the efficiency of clinical rehabilitation methods in dysphagia patients. In addition, these methods have some other certain limitations such as radioactive, complex operation, high cost, and invasive risk, which would restrict their clinical application for the screening and evaluation of swallowing functions. Myoelectric signal is an electrophysiological response of muscular activities. Surface electromyogram (sEMG), which is the myoelectric signal noninvasively acquired by the electrodes on skin surface has been widely used in a variety of clinical and biomedical applications [18]. Since swallowing is essentially a semiautomatic motor action of muscles, the sEMG has been also used to assess the swallowing functions [19], which should outperform radiographic, manometric, and endoscopic techniques for the study of deglutition. A number of previous investigations have been conducted to evaluate the swallowing functions by using sEMG recordings [20–26]. McKeown et al. used the independent component analysis method to analyze the sEMG signal measured from the neck region of several subjects to examine the muscle activation patterns during swallowing [23]. Vaiman and Eviatar recorded sEMG signals from a set of healthy children to establish a database for specific muscular activities during swallowing, and proposed the usage of sEMG technique for the assessment of patients with swallowing disorders [24, 25]. Perlman et al. explored the durations and temporal relationships of electromyographic activities with bipolar surface electrodes from muscles during swallowing of different boluses, and found that interarytenoid muscle showed a significant difference in duration between the saliva and 10 ml of water [26].

Note that most previous studies used sEMG recordings from one individual muscle or a pair of muscles to investigate the muscle activities during swallowing. The sEMG signals from a few EMG sensors (1–4 electrodes) can give us some electrophysiological information about the characteristics of the muscle contractions related to deglutition. However, a complete deglutition requires a complex sequence of oral and pharyngeal events for the passage of a bolus into the esophagus. The sEMG signals only from several sparse sensors might not physiologically provide enough dynamic properties of a whole deglutition procedure when one complete swallowing would involve the activities of a number of muscles and cranial nerves. Therefore, dynamically and completely exploring the electrophysiology of deglutition-related muscles should be vital for the assessment of swallowing functions and disorders such as dysphagia.

High-density sEMG (HD sEMG) is non-invasive technique to measure electrical muscle activity with a large number of closely spaced electrodes overlying an area of the skin [27, 28]. The method of HD sEMG have been utilized in a number of studies to obtain fine electrophysiological information on activities of muscles by using a two-dimension (2D) electrode array [29–34]. While the single-channel recording methods could disregard spatial distribution of the skin potential, the HD sEMG technology offers a new approach to obtain the temporal and spatial properties of the electrical muscle activities [35, 36]. The HD sEMG have been used in several different studies such as diagnosing neuromuscular disorders [30], estimating muscle conditions [32–34], and controlling a human–machine interface [36]. The feasibility of using HD sEMG dynamic topography to continuously visualize muscle activities of normal swallowing has been investigated in a pilot study conducted in our laboratory [37]. Our preliminary results suggested that sEMG recordings could provide the useful information about the electrophysiological characteristics of swallowing muscles. Note that only three subjects were used and just two cases, dry swallowing and swallowing a cup of water, were examined in the pilot study.

In the current study, the performance of using HD sEMG technique to physiologically evaluate swallowing functions would be systemically investigated when a number of subjects were swallowing different kinds of eatable materials. Since understanding the functions of a normal deglutition should be an essential prerequisite for early diagnosis and proper therapy of dysphagia, this study would concentrate on the evaluation of normal swallowing functions by using dynamic HD sEMG. sEMG signals generated by muscle activities during swallowing were continuously recorded with a HD two-dimension (2D) electrode array. The sEMG recordings from an entire swallowing process were properly segmented into a series of sEMG analysis windows and then the sEMG energy of each analysis window was computed to generate a 2D map. These sEMG energy maps could visually demonstrate the muscle activities in deglutition since they will present the electrophysiological responses of voluntary and involuntary muscle contractions. Some quantization parameters were calculated from each of the sEMG energy maps and used to objectively and quantitatively examine the cooperation of different muscles during the swallowing. The HD sEMG approach proposed by this study may be useful for the continuous visualization of muscular activities and accurate quantification of muscle energy distribution related to normal swallowing processes, which might provide a potential tool for screening swallowing disorders to assist precise treatment of dysphagia patients.

## Methods

### Subjects

In this study, twelve subjects (five females and seven males) with normal swallowing capabilities were recruited. All subjects were examined by an occupational therapist to make sure that they had no history of dysphagia or any other medical problems that might affect swallowing. The mean age of the subjects was 27 years with a range of 23–38. The protocol of this study was approved by the Institutional Review Board, and the procedures were performed in accordance with the ethical standards of the committee on human experimentation at the Shenzhen Institutes of Advanced Technology, Chinese Academy of Sciences. Before the study, the subjects received a complete explanation of the purpose, risks, and procedures of the investigation. All the subjects gave written informed consent and provided permission for publication of photographs with scientific and educational purposes.

### HD sEMG signal acquisition

**Fig. 1:**
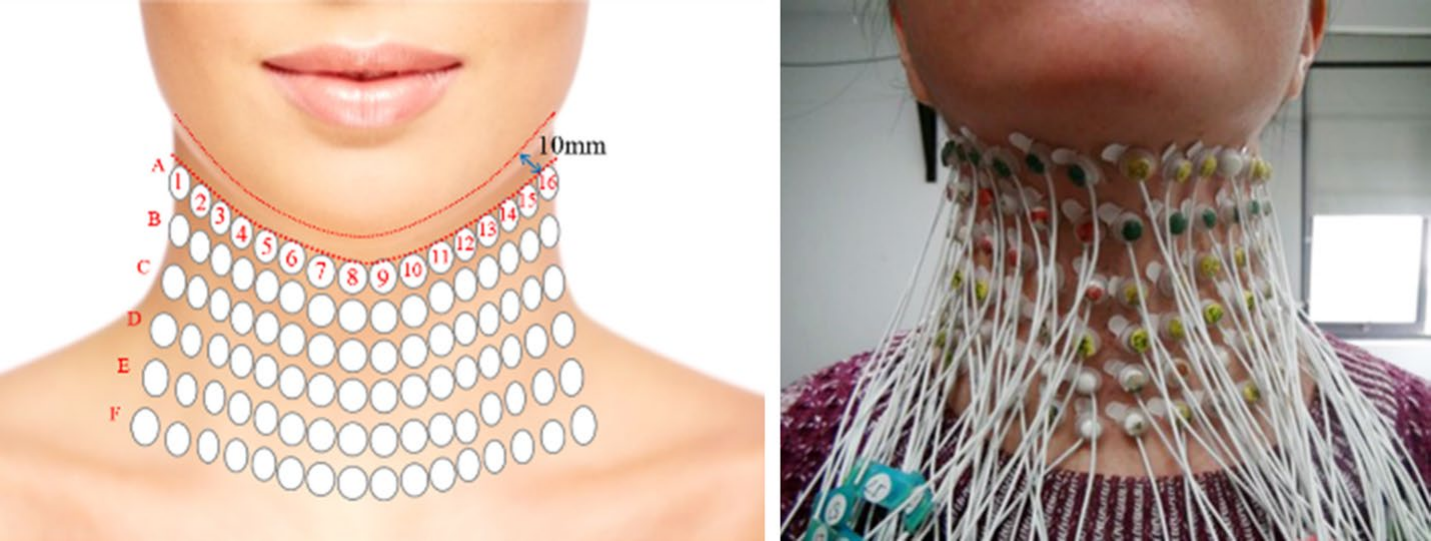
Placement of high-density electrode array on the front neck

The HD sEMG signals were acquired from all the enrolled subjects. Firstly, each electrode was properly cleaned by using alcohol pad and then coated with a conductive gel to ensure good adhesiveness and conductivity between the electrode and the skin. Secondly, the skin surface where the HD sEMG electrodes located was carefully cleaned with alcohol swabs to remove dry dermis and skin oils that could degrade the quality of the sEMG recordings. Finally, a 2D electrode array made up of 96 channels was evenly aligned and placed on the front region of the neck (covering submental and infrahyoid muscle complexes) (Fig. 1) for recording muscular signals during various swallowing tasks.

In order to obtain comprehensive electromyography information, sufficient electrode number and small inter-electrode distance is necessary. To avoid the collision of two adjacent electrodes during swallowing tasks, a certain distance should be kept between neighboring electrodes. Correspondingly, the 96-channel electrode array structured in a 6 × 16 grid equally spaced with an interval of 15 mm between two adjacent rows (A–F) and columns (1–16) was selected so that the front neck could be fully covered for all subjects (Fig. 1). Each electrode has a circular silver-plated recording surface with an external diameter of 10 mm. The first row (Row A) of sixteen electrodes was placed about 10 mm below the lower jaw to facilitate the positioning of the electrode array, and the electrodes of A8 and A9 were placed around the center of the submental triangle for locating purpose. Then the other five rows were placed downward relative to Row A. In addition, a reference electrode was placed on the right wrist of each subject to serve as the ground for all electrodes. After the electrode array was placed properly, HD sEMG signals were filtered with a band-pass filter (10–500 Hz) and recorded simultaneously from all the 96 channels by the REFA 128-channel system (TMSi International, the Netherlands) at a sampling rate of 1024 Hz.

### Experimental procedures

**Fig. 2:**
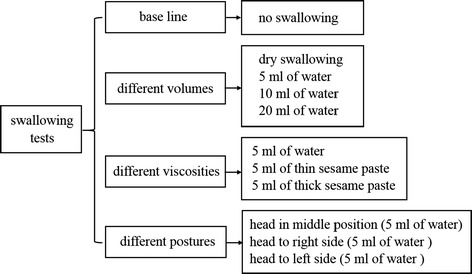
Four different swallowing tests and the corresponding swallowing tasks

In the experiment of HD sEMG acquisition, a subject was seated on a comfortable chair and instructed to complete different swallowing tasks. Four different swallowing tests (Fig. 2) were adopted in the study to systemically evaluate the muscle activities of an entire swallowing procedure by using HD sEMG signals. For all the subject, sEMG were firstly acquired when they did not perform any swallowing action, which were used as a base line for sEMG normalization. Secondly, the subjects were asked to drink different volumes of water with a normal swallowing speed. The voluntary swallowing test had four successive tasks: swallowing saliva (dry swallowing) and drinking 5, 10, and 20 ml of water at a time, respectively. Thirdly, a swallowing test to drink different viscous materials was conducted, where each subject was asked to sequentially swallow 5 ml of bolus with different viscosities: water, thin sesame paste, and thick sesame paste. The thin sesame paste was made of 100 ml of water and 20 g of sesame powder, while the thick sesame paste was mixed by 100 ml of water and 35 g of sesame powder. During the first three swallowing tests, the subjects were required to keep their face forward and avoid any body movements. Finally, in order to examine the effect of head posture changes during swallowing on the sEMG recordings, the subjects were asked to retain their head at middle position, to right side, and to left side when drinking 5 ml of water, respectively. For all the four swallowing tests, each swallowing task was repeated for three times. When performing a swallowing task, subjects were required to hold the water or sesame bolus in their mouth for a while and then to get started swallowing normally. HD sEMG signals were recorded from 96 channels during the entire process of all the swallowing tasks and stored locally for further offline analysis.

### Data analysis

#### sEMG energy maps

The HD sEMG signals acquired during different swallowing tasks were properly filtered by using digital filters to reduce the impacts of ECG artifacts picked up by the electrodes and other baseline variations caused by unintentional body movements of the subjects. Then the sEMG recordings from an entire swallowing process were segmented into a series of sEMG analysis windows with a window length of 100 ms for each channel. For each sEMG analysis window, its root-mean square (RMS) value was computed for all the 96 channels as follows, which represented the averaged power (energy) of EMG signals over the analysis window.

1 $$I_{i,j}^{a} = \sqrt {\frac{{\sum\nolimits_{n = 1}^{N} {X_{i,j}^{2} (a,n)} }}{N}} \;$$

where $I_{i,j}^{a}$ is the RMS value of sEMG from the channel located at *row i* and *column j* of the 2D electrode array in *a* *th* analysis window, *X*_*i*,*j*_ (*a, n*)is the sEMG amplitude of the channel (*i, j*) in *a* *th* analysis window, and *N* is the total number of sEMG samples *N* is the total number of samples in the analysis window. Therefore, a 2D RMS matrix with a dimension of 16 × 6 would be obtained for each of the analysis window. Each 16 × 6 RMS matrix was extended by using a spline interpolation algorithm and then the extended matrix was used to build a 2D sEMG energy color map, where the intensity values were represented by color tones scaled linearly between maximum (hot) and minimum (cold). By displaying the map slide by slide over time, these topographical maps would graphically demonstrate the dynamic electrophysiological activities of muscles in an entire swallowing process. The dynamic sEMG maps of the entire swallowing process were divided (by time) into five consecutive non-overlapped intervals, in accordance with the five physiological phases by other studies, so that the motions of swallowing structures of each time interval could be known according to the literature [38]. Then the RMS values within each interval were calculated and the energy map of each interval was projected onto the human neck area to exhibit the energy distribution of each swallowing phase.

#### Quantitative analysis

**Fig. 3:**
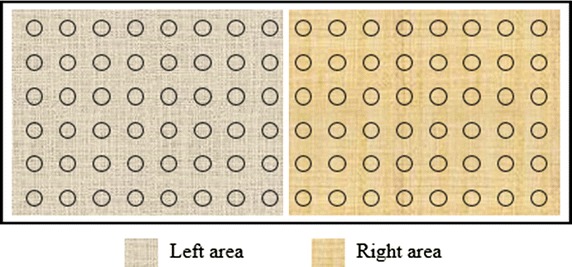
The left and right areas of electrode array to evaluate the symmetry of muscle activities

In the study, some quantitative indices were proposed to evaluate the electrophysiological characteristics of swallowing muscles. The 2D electrode array were equally divided into two sections from the center line of the neck: left-lateral electrodes and right-lateral electrodes, as shown in Fig. 3. Each section contained a total of 48 electrodes (8 × 6 array). The mean values of sEMG RMS were calculated by averaging the RMS values over all the electrodes (48 electrodes) of the left and right sections and over all the analysis windows in an entire swallowing process, respectively. Two-tailed t tests were used to test whether the mean LRER values averaged across all subjects were significantly different from 1.0 for different tasks, with a *p* > 0.05 indicating that there was no significant difference between the left and right sides in muscle activities. Two-tailed t-tests were also used to examine whether the averaged LRED values were significantly different from 0, with a p > 0.05 meaning nearly equivalent energy between the two sides. The mean RMS values were designated as *mRMS*_*left*_ for the left electrode section and *mRMS*_*right*_ for right electrode section. A quantitative index, named as the Left/Right Energy Ratio (*LRER*), was defined to measure the symmetric features of left and right neck muscles in deglutition as follows:

2 $$LRER = \;\frac{{mRMS_{left} }}{{mRMS_{right} }}$$

In addition, another quantitative index, called as Left/Right Energy Difference (LRED), was proposed and used to assess the symmetry of muscle activities on the left/right sides. LRED was defined as the absolute difference between the sum of left and right RMS values as follows.

3 $$LRED = \frac{{sum(RMS_{left} ) - sum(RMS_{right} )}}{{\frac{1}{2}sum(RMS_{N} )}} \times 100\%$$

where *sum*(RMS_*left*_) and *sum*(RMS_*right*_) was the summation of the RMS values obtained from the left and right EMG electrode section, respectively, and *sum*(RMS_*N*_) represented the summation of the RMS values of all the EMG electrodes.

## Results

### Dynamic sEMG energy maps during a normal swallowing

**Fig. 4:**
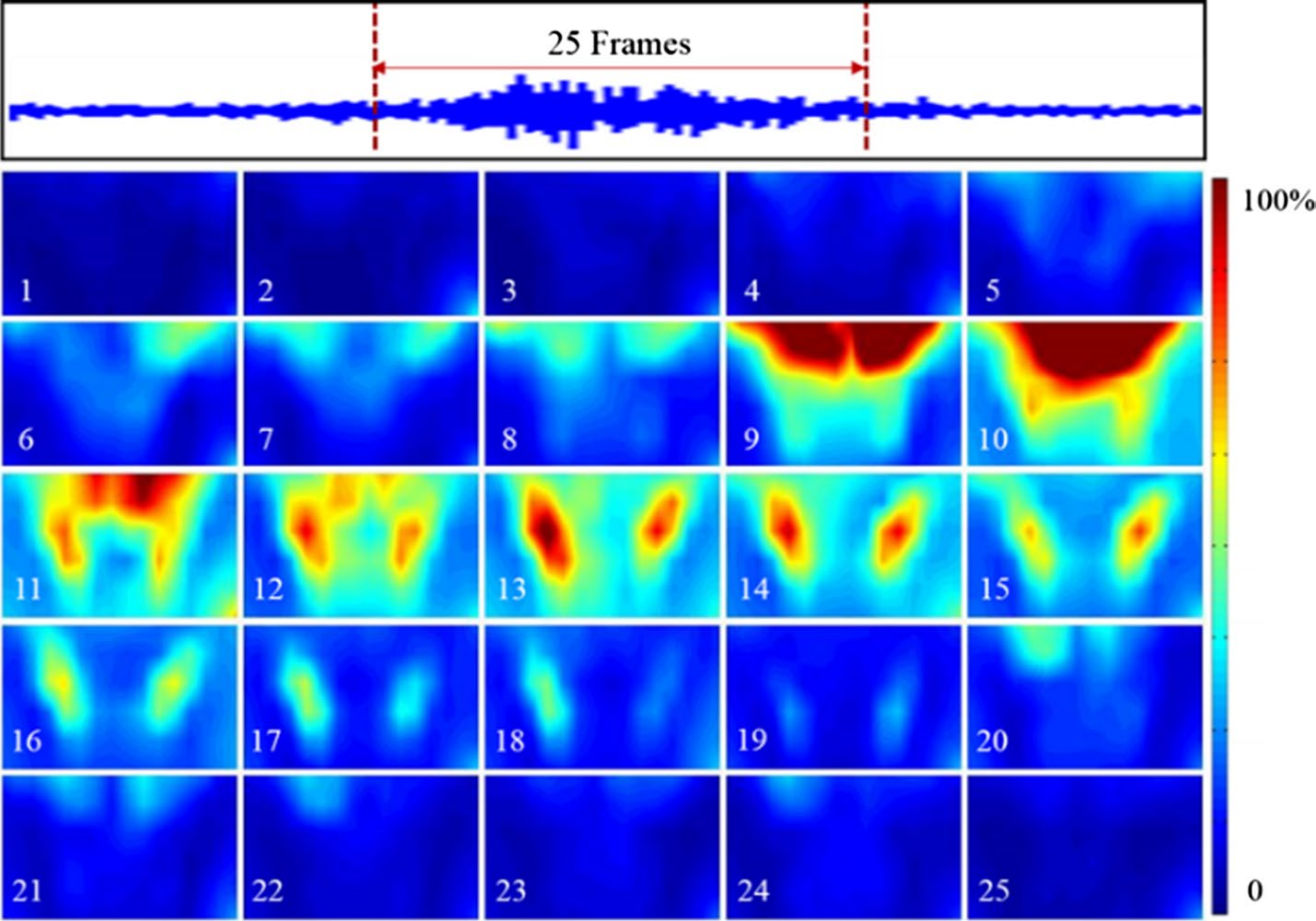
Dynamic HD sEMG energy maps when swallowing 5 ml of water

In this study, sEMG signals were recorded during different swallowing tasks, and the dynamic sEMG maps of each swallowing task were constructed for all the subjects. A typical example of 2D sequential sEMG energy maps (25 frames) was shown in Fig. 4 when a subject was swallowing 5 ml of water. Different myoelectrical energy intensities were mapped with different colors in the energy maps and the maps were globally normalized with the maximum RMS value in all the 25 frames.

From Fig. 4, we can observe that the energy maps showed dynamic myoelectrical activities of the neck muscles during a normal swallowing process. At the beginning (frames #1 to #4), the subject held water in his mouth and there was almost no any active region of the muscles. When the subject was instructed to start swallow the water bolus, the muscular activities started to emerge from the top edge (frame #5) and then gradually spread downward to reach a maximum at the 10th frame. From frame #11 to #12, the EMG activity areas on the maps started splitting up to two parts from the center line. Later, from frame #13 to #17, the maps clearly presented an approximately symmetric activity regions of muscle contractions on the left and right sides. And the overall intensity of muscle activities on the both sides reached a maximum value on frame #13. Thereafter, the two symmetric activity areas moved down and their intensity decreased gradually on the consecutive frames (frame #14 to #17). Afterwards (frame #18 to #20), the regions of the muscular activities traveled upwards back to the upper locations. In the end of swallowing, EMG activities diminished gradually in the center of maps and finally disappeared in frame #25. The similar patterns of muscle activities on dynamic sEMG maps could also be observed on other twelve subjects when they were swallowing water.

**Fig. 5:**
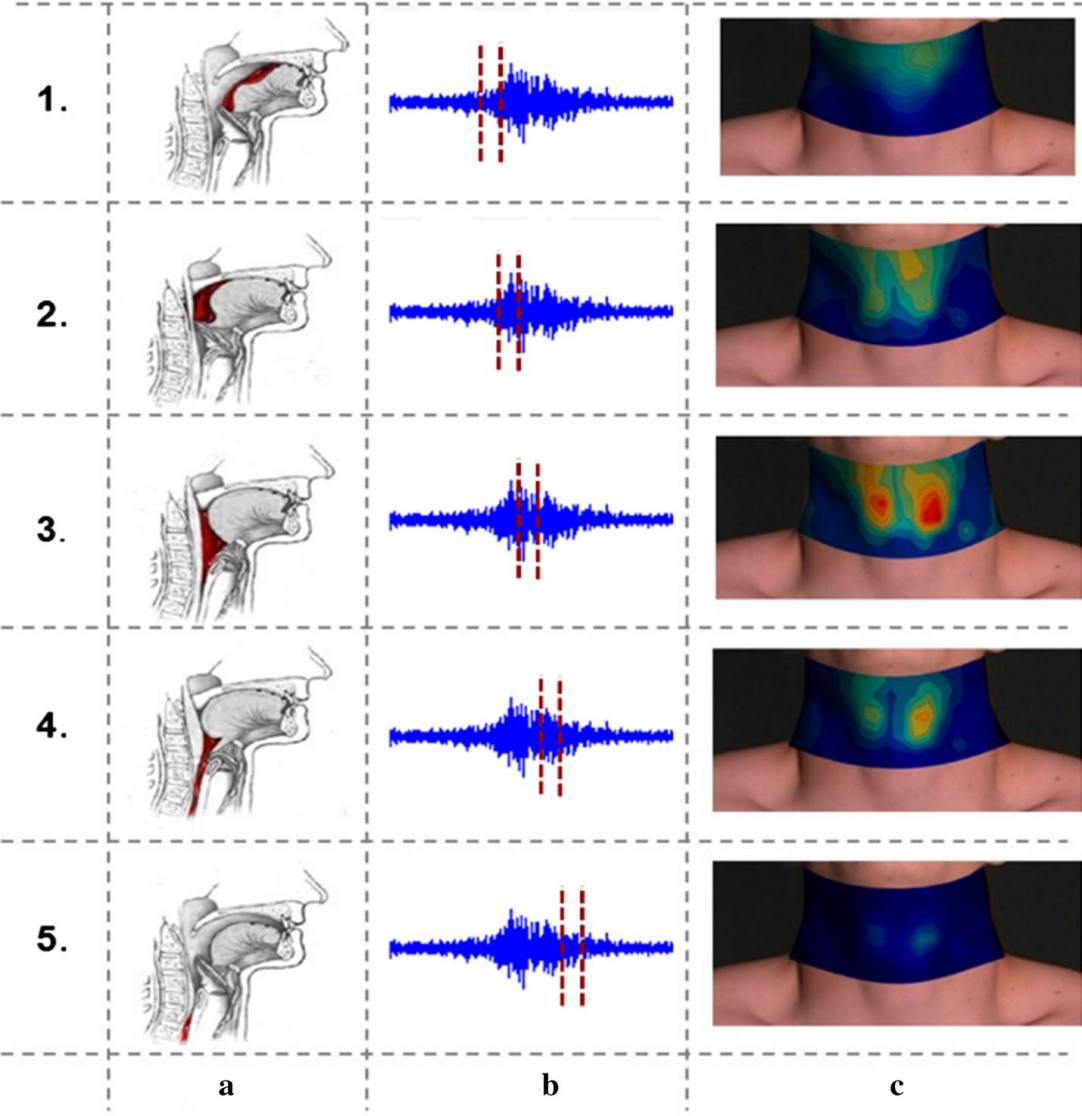
**a** Five physiological phases of the swallowing process; **b** sEMG signal and the time interval of each phase; **c** averaged sEMG energy maps of each phase when swallowing 5 ml of water

According to the literature [38], a normal physiological process of swallowing can be divided into five phases, as shown in Fig. 5a. The time interval of each phase is marked and labeled by two vertical lines on sEMG signal recorded from the submental muscle (Fig. 5b). The RMS values of the five continuous intervals in each phase were averaged to create an averaged map for each phase as shown in Fig. 5c. This typical normal sEMG energy maps showed the pattern with symmetric muscle activity on both left and right sides, and the sEMG energy intensity in the submental and infrahyoid area increased or decreased in an orderly manner throughout the entire duration of swallowing process. The pattern of muscle activations on these dynamic sEMG maps was associated with physiological and biomechanical mechanisms of swallowing muscles for better understanding of the actual swallowing process. In the beginning of a swallowing, i.e. in phase 1, the subjects were instructed to swallow water bolus in their mouth, where the soft palate elevated and the bolus was propelled posteriorly toward the pharynx. The activities of the infrahyoid muscle decreased, and the ones of the submental muscle increased responding to the lifting of the bolus. Accordingly, the high intensity concentrated on the top of the maps (frame #1 in Fig. 5c). In phases 2–3, the bolus was pushed further into the pharynx by the sequential contraction of pharyngeal constrictor muscles. In sEMG topographies (frame #3 in Fig. 5c), two areas of the highest intensity appeared symmetrically in both sides of larynx. In phases 4–5, with the relaxation of the cricopharyngeal muscle and the opening of the upper esophageal sphincter, the bolus passed into the esophagus. Thus the intensity on sEMG maps decreased in the center area and returned to the rest level. For sEMG swallowing maps, the frame with the increased intensity on the top of the map could be regarded as the onset of a swallow and the frame with the decreased intensity in the center of the map as the offset, and therefore an individual swallowing process could be identified with these two key frames, as shown in frames #1 and #5 of Fig. 5c.

Generally speaking, when the subjects were swallowing different volume of water once, i.e., 0 ml (dry swallowing), 5, 10, and 20 ml, respectively, the sEMG energy maps illustrated similar dynamic temporal trend as those when swallowing 5 ml of water as shown in Figs. 4 and 5. However, there were some slight difference among swallowing different volume of liquid. The sEMG energy of muscle activities gradually strengthened with the increase of swallowing volume of water. A dry swallowing process seemed to take a longer time to complete one swallowing than swallowing water. Note that these results of the current study were coincident with those of our pilot study conducted on three normal subjects [37], thus the results from swallowing different volumes of liquid were not presented in the paper.

### Effects of viscosity on swallowing muscle activity

**Fig. 6:**
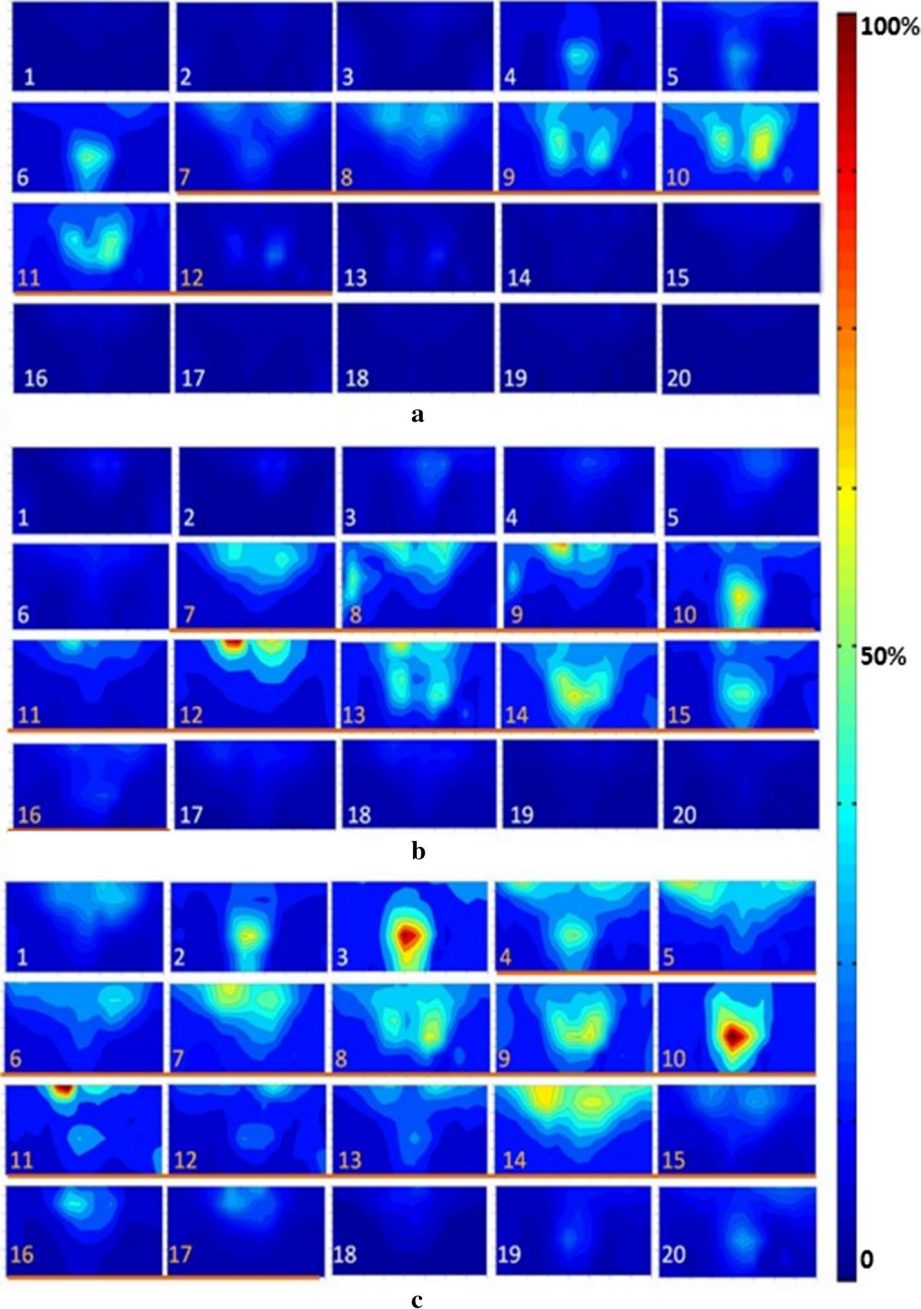
The dynamic HD sEMG energy maps when swallowing bolus with different viscosities. **a** Water; **b** thin sesame paste; **c** thick sesame paste

The effects of bolus viscosity on normal swallowing process were also investigated by using the HD sEMG energy maps. Figure 6 showed the dynamic sEMG energy maps when one subject was swallowing a 5 ml of bolus with three different viscosities (water, thin sesame paste, and thick sesame paste). For each swallowing task, twenty consecutive frames of sEMG maps were constructed using the averaged RMS values in twenty consecutive non-overlapped epochs. It can be seen from Fig. 6 that the sEMG energy maps from the three swallowing tasks revealed explicit difference with regard to the duration and strength of the swallowing process, but similar distribution pattern of muscle activities. When swallowing water, one swallowing cycle could be clearly observed on the sEMG maps (frame #7 to #12, Fig. 6a) and the intensity of sEMG energy reached the maximum in frame #10. When swallowing thin and thick pastes, two swallowing cycles were observed in Fig. 6b, c. The first swallowing cycle was recognized from frame #7 to #11 and then the second one was observed from frame #12 to #16. The actual swallowing duration increased significantly with the increased viscosities of the food categories from liquid to thin paste and to thick paste. For a complete swallowing process, the muscle activities would last about 6 frames (from #7 to #12, Fig. 6a) for swallowing liquid, about 10 frames (from #7 to #16, Fig. 6b) for swallowing thin paste, and 15 frames (from #4 to #18, Fig. 6c) for swallowing thick paste. The maximum sEMG activities increased significantly from swallowing liquid to thin paste and from thin pastes to thick pastes for both submental and infrahyoid areas. For thick pastes, the peak activity appeared in the center of maps and reached the maximum, as shown in the frame #10 of Fig. 6c.

### The effects of head posture on swallowing muscle activity

**Fig. 7:**
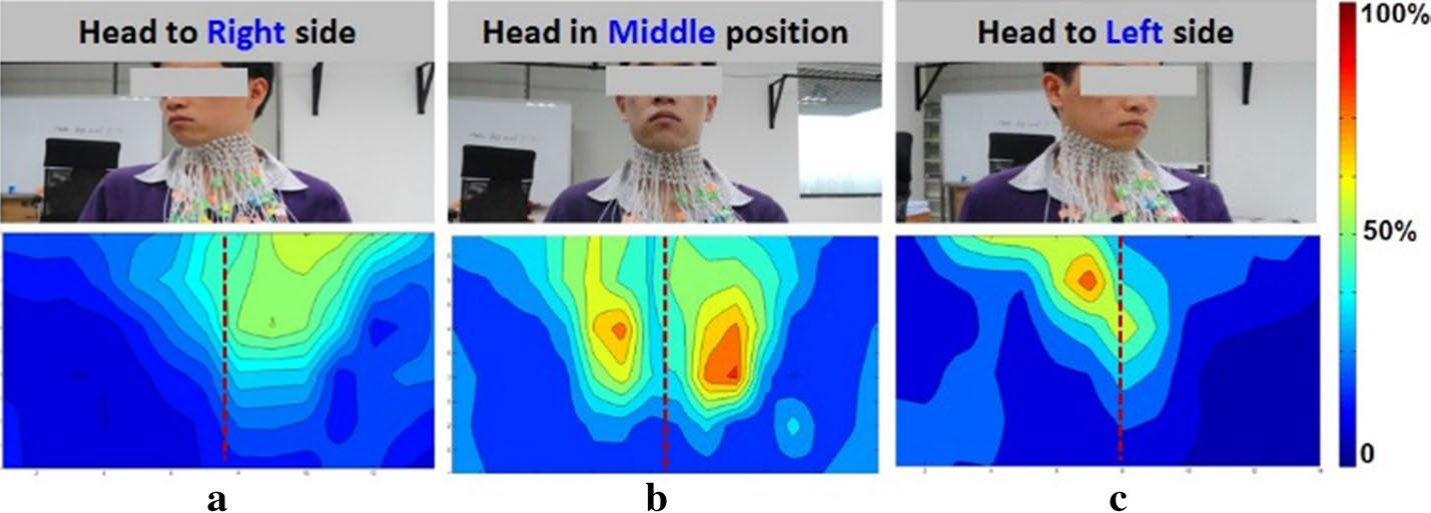
The dynamic sEMG energy maps when swallowing with three different head postures. **a** Head to right side; **b** head in middle position; **c** head to left side

Since postural change have been used in clinical context to compensate for dysphagia [39], the effect of posture change on swallowing processes was also investigated by dynamic HD sEMG maps in this study. Figure 7 shows the typical averaged sEMG energy maps when a subject was swallowing 5 ml of water with different head postures: head to left and head to right. The sEMG energy maps corresponding to each head posture were represented by one single frame in the middle of the swallowing. It can be seen from Fig. 7 that the muscular activities were rather symmetrical between the left and right side when the head was in the middle (forward) position, as shown in Figs. 4, 5 and 6. When the subject turned his head to one side (either left or right side), an outstanding change in the energy map was that the concentration of the muscular activities shifted to the other side in oppose to the head position, i.e., the muscles on the left were more active when the subject’s head was kept to the right side (Fig. 7a) and vice versa (Fig. 7c). Moreover, evident decrease in the amplitude of the muscular activities was also observed when the head was turned to either side, compared with the case of head in the middle position (Fig. 7b).

### Quantitative analysis

**Fig. 8:**
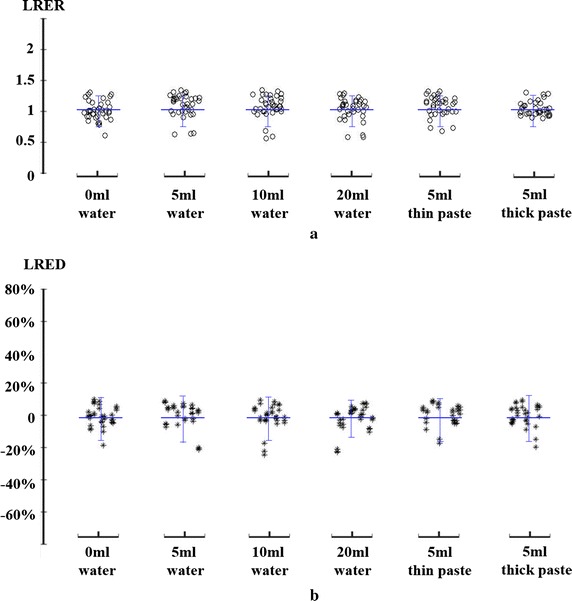
Comparison of parameters of LRER **a** and LRED **b** grouped by different swallowing tasks to evaluate the symmetry of the left and right sides

During a normal swallowing process, one important characteristics on the dynamic sEMG energy maps is symmetric neck myoelectric activities. To quantitatively measure the symmetric characteristics of swallowing muscle activities, two quantitative parameters, LRER and LRED, were calculated from the dynamic HD sEMG energy maps. Figure 8 shows the scatter plot of LRER and LRED of all the subjects grouped by different swallowing tasks. It could be observed from Fig. 8 that the LRER parameter from all the subjects were closely distributed and the mean values were not significantly different from 1.0 (p > 0.05) for all the swallowing tasks, as shown in Fig. 8a. Further analyses by employing the LRED parameters illustrated that the normalized energy difference between the left and right sides were not significantly different from 0 (p > 0.05) with a standard deviation of 0.07 across all the swallowing tasks (Fig. 8b). A few dispersed points away from the mean of LRED might be attribute to random noises or unintentional body movements.

## Discussion

The primary objective of this work was to investigate the feasibility of HD sEMG maps as a new approach for the continuous visualization of swallowing process. This objective has been reached by using a 2D electrode array on neck muscles to record the sEMG during swallowing. In the current study, the HD sEMG signals were recorded with an array of closely-spaced electrodes, and were processed in the space dimension to assess global muscular activities related to swallowing. The RMS value of each channel over a time duration was calculated and was considered as a pixel located at the definite position of the electrode array. Thus a series of 2D temporal sEMG maps were constructed by the RMS values of 96 channels. The dynamic HD sEMG maps would provide both spatial and temporal properties of the electrical muscle activity and presented the muscle contraction coordination that would be closely related to the swallowing function. The distribution of sEMG intensities were visually inspected on the maps where the intensity values were represented by color tones (cold to hot color). Thus the HD sEMG maps would provide a quantification of the temporal and spatial properties of the electrical muscle activity. This technique could overcome some limitations of the previous studies that were only based on the sEMG signals recorded with a few electrodes located in an individual muscle or a muscle pair.

In this paper, the normal swallowing process has been systematically investigated by the HD sEMG signals with an attempt to establish a dynamic muscle activity pattern of normal swallowing, which might be served as a reference for screening and assessment of swallowing disorders or dysphagia in the future studies and possible clinical applications. Our results from a number of normal subjects showed that the dynamic swallowing topographic maps could present the changing process of sEMG spatial distribution during swallowing, providing a new method to analyze the muscle contraction coordination during swallowing. The sEMG potential maps mainly reflected the activities of two muscle groups: submental and infrahyoid muscles, which corresponded to the high intensity on the top and in the center of maps, respectively. Our results were consistent with those of previous studies. A swallowing process could be characterized by submental and infrahyoid muscle bursts, initiated by increased submental muscle activity and terminated by decreased infrahyoid muscle activity.

During a normal swallowing, the bolus would be transported from the oral cavity though the pharynx to the esophagus. Accordingly, the temporal sEMG energy concentration in the HD maps was in a consistent shift with the movement of the bolus. At the beginning of the swallowing task, the water was hold in the oral cavity and sitting on the dorsum of the tongue, thus the submental muscles became active and a high intensity region appeared at the top of the map. When the water was propelled from the oral cavity into the oropharynx, the energy concentration shifted downward to the region of infrahyoid muscles, accomplished by evident increase of activity amplitude. Later the larynx, together with the nasopharynx, opened accordingly resulting in a maximum in the energy map. Finally, when the water was passed down to the esophagus, the muscle activities started to decrease until a resting state was reached. The dynamic HD sEMG maps could provide spatial and temporal properties of the muscle electrical activity, and visualize the muscle contraction that closely related to the swallowing function. The HD sEMG maps could be served as a new approach for the continuous visualization of the swallowing process, and could be possibly used as a potential tool for fast screening and objective assessment of swallowing disorders or dysphagia.

The entire swallowing process can be divided into five sub-phases in normal physiological process of swallowing ease detailed analysis of swallowing [38]. In this study, five HD sEMG energy maps averaged within the time interval of each phase were constructed to show the swallowing phases corresponding to the physiological process of swallowing (Fig. 5). In phase 1, the subjects were instructed to swallow the bolus in their mouth, so the tip of the tongue moved upward and the bolus was propelled posteriorly toward the pharynx. As a result, the activities of the infrahyoid muscles decreased and those of the submental muscles increased in response to the lifting of the bolus, indicated by a high intensity region on the top of the energy map (frame #1 in Fig. 5c). In phase 2, the bolus was pushed into the oropharynx while the area of soft palate and larynx began to elevate. Consequently, the submental muscle activities got stronger, and the region with outstanding muscle activities started to expand and moved down to the center (frame #2 in Fig. 5c). In phase 3, the hyoid bone and larynx were pulled upward, and the bolus was squeezed downward through the oropharynx and further into the hypopharynx by the sequential contraction of pharyngeal constrictor muscles. So muscle activities became more intense and concentrated in the center of the map (frame #3 in Fig. 5c). Meanwhile, the energy concentration split into two symmetric areas on the left and right sides of the larynx, meaning that the energy of the swallowing activities had a nearly equivalent distribution on both sides. In order to quantitatively analyze the symmetry properties of both sides, two metrics (LRER and LRED) were calculated and the results showed strong consistency for all subjects and different swallowing tasks (with the head in the middle position). These results revealed that the muscle associated with swallowing located at the left area and the right area of the neck were activated simultaneously with nearly equivalent intensity and durability, thus producing a balanced oral pressure to aid the movement of the pharynx and larynx in normal swallowing process. This symmetry analyses were consistent with our previous study [40]. Finally, in phase 4 and 5, the cricopharyngeal muscle and the upper esophageal sphincter relaxed, as the bolus passed into the esophagus. Thus, the overall intensity of the energy maps gradually decreased and down to the resting-state level. The HD sEMG energy maps were well associated with the physiological and biomechanical principles of swallowing, making the HD sEMG method a simple and non-invasive tool to accurately differentiate phases of the swallowing. It could also help researchers and therapist better understand the dynamic muscular activities associated with each phase of normal swallowing process. Additionally, the HD sEMG energy maps was used to examine the effects of different volumes, viscosities, and head postures on normal swallowing process. The energy maps obtained from swallowing water with different volumes showed that the duration of the dry swallowing was the longest across the four volume tasks. This might be attributed to the lubrication effects of water that makes swallowing easier, thus the water swallowing took shorter time than try swallowing. Moreover, the muscular activities slightly increased when the volume of the water increased. These findings were in accordance with results from the previous studies [41–43].

The energy maps obtained from swallowing bolus of different viscosities (water, thin paste, and thick paste) showed that the time it took to complete the swallowing significantly prolonged when the viscosity increased (Fig. 6). This prolongation was usually accompanied by the increase in the number of swallowing attempts as observed in Fig. 6b, c. It suggests that multiple of retries would appear if the subjects found the bolus difficult to swallow. For the swallowing of bolus with increased viscosity, evident increase in the amplitude of muscular activities were also observed (Fig. 6). This might be attribute to larger oral pressure and stronger laryngeal elevation when swallowing bolus with higher viscosity. Similar findings were also reported in a number of previous studies that evaluated swallowing processes by using other techniques [7, 44–47], and could provide much more details because of the usage of the HD sEMG. The results supported that increasing bolus viscosity required larger motor neuron pools and resulting more muscular activities. The HD sEMG energy maps of different volumes and viscosities implied that clinical practice should consider about the influence of bolus material and volume to manage the diet of dysphagia for reducing secondary physiological impairment.

The energy map in Fig. 7 revealed the pattern changes of the swallowing process when the head was kept at different postures. When the subject turned his head to the left side, the bolus pathway shifted to the right side so the muscles of the right side became more active, indicated by more energy concentration on the right side in the HD maps. Similarly, the major muscular activities gathered at the left side when the head was turned to the right side. These findings agreed with previous study [40, 48] and could provide a potential method to improve swallowing in patients with unilateral dysphagia.

The purpose of present study was to evaluate the ability of dynamic sEMG topography to represent the process of normal swallowing. In the further study, the patients with different types of swallowing disorders will be recruited, and the muscle activity patterns of normal swallowing obtained in this study might be a reference for further investigation focused on the development of clinical application for the screening and/or evaluation of swallowing function or dysphagia.

## Conclusions

In this study, the method of HD sEMG energy maps was proposed to visualize the continuous dynamic process when the subjects were swallowing bolus of different volume and viscosity. The results showed that a dry swallowing process seemed to take a longer time than water swallowing. The maximum sEMG activities increased significantly from swallowing liquid to thin paste and from thin pastes to thick pastes for both submental and infrahyoid areas. This study suggested that the muscular activities were nearly symmetrical between left and right sides for different swallowing tasks when the subject’s head was kept in the middle position. The energy maps of different head postures revealed that the concentration of the muscular activities shifted to the other side in oppose to the head position. The proposed method might provide a useful tool to evaluate the normality of the muscular functions, and help to pin out the possible cause of swallowing difficulties for patients with swallowing disorders.

## Abbreviations

HD sEMG: high-density surface electromyography
2D: two-dimension
RMS: root-mean square
LRER: Left/Right Energy Ratio
LRED: Left/Right Energy Difference
